# Mental health and cultural continuity among an urban Indigenous population in Toronto, Canada

**DOI:** 10.17269/s41997-022-00709-6

**Published:** 2022-12-16

**Authors:** Michelle Firestone, Stephanie McConkey, Emily Beaudoin, Cheryllee Bourgeois, Janet Smylie

**Affiliations:** 1 MAP Centre for Urban Health Solutions, Unity Health Toronto, Toronto, ON Canada; 2 Dalla Lana School of Public Health, University of Toronto, Toronto, ON Canada; 3 Well Living House, St. Michael’s Hospital, Unity Health Toronto, Toronto, ON Canada; 4 Department of Epidemiology, Dalla Lana School of Public Health, University of Toronto, Toronto, ON Canada; 5 Seventh Generation Midwives Toronto, Toronto, ON Canada

**Keywords:** Indians, North American, Culture, Community-based participatory research, Mental health, Indiens d’Amérique du Nord, culture, recherche participative communautaire, santé mentale

## Abstract

**Objectives:**

Mental health and psychiatric disorders significantly affect individuals on personal and social levels. Indigenous populations in Canada have disproportionately high rates of mental health diagnoses. Our Health Counts (OHC) Toronto assessed mental health, racism, family disruption, and cultural continuity among urban Indigenous people. The objectives of this study were to understand (1) the demographics and characteristics of Indigenous adults with a diagnosed psychological/mental health disorder and (2) potential associations of psychological/mental health diagnoses with experiences of colonization and cultural continuity among Indigenous adults in Toronto.

**Methods:**

Using community-based participatory research methods, Indigenous adults in Toronto were recruited by respondent-driven sampling (RDS) to complete a comprehensive health assessment survey. RDS-II weights were applied to calculate population-based estimates, and adjusted odds ratios with 95% confidence intervals were produced using logistic regression, controlling for age and gender.

**Results:**

Among Indigenous adults, nearly half (45%) reported receiving a mental health diagnosis. Participants reported lifetime anxiety disorders (53%), major depression (51%), and high rates of suicide ideation (78%). Of Indigenous adults with a diagnosed mental health disorder, 72.7% reported participating in ceremony. Attending residential schools (OR: 7.82) and experiencing discrimination (OR: 2.69) were associated with having a mental health disorder.

**Conclusion:**

OHC Toronto responded to the gaps in health assessment data for urban Indigenous people. Despite historic and ongoing trauma, Indigenous people have maintained cultural practices and a strong sense of identity. Efforts aimed at supporting Indigenous well-being must respond to the roots of trauma, racism, and existing Indigenous community knowledge and strengths.

## Résumé

**Objectifs:**

Les troubles psychiatriques et de santé mentale ont des effets considérables sur le plan personnel et social. Les populations autochtones du Canada ont des taux démesurément élevés de diagnostics en santé mentale. L’organisme Our Health Counts (OHC) Toronto a évalué la santé mentale, le racisme, les perturbations familiales et la continuité culturelle de personnes autochtones en milieu urbain. L’étude visait à comprendre 1) le profil démographique et les caractéristiques d’adultes autochtones ayant un trouble psychologique ou de santé mentale diagnostiqué et 2) les associations possibles entre les diagnostics psychologiques ou en santé mentale et les expériences de colonisation et de continuité culturelle chez les adultes autochtones de Toronto.

**Méthode:**

À l’aide de méthodes de recherche participative communautaire, des adultes autochtones de Toronto ont été recrutés par échantillonnage en fonction des répondants (EFR) pour répondre à une enquête d’évaluation globale de la santé. Des pondérations EFR-II ont été appliquées au calcul d’estimations populationnelles, et des rapports de cotes ajustés avec des intervalles de confiance de 95 % ont été produits par régression logistique, après avoir apporté des ajustements pour tenir compte des effets de l’âge et du sexe.

**Résultats:**

Chez les adultes autochtones, près de la moitié (45 %) ont déclaré avoir reçu un diagnostic en santé mentale. Les participants ont déclaré avoir connu au cours de leur vie des troubles anxieux (53 %), une dépression majeure (51 %) et, dans une proportion élevée, des pensées suicidaires (78 %). Chez les adultes autochtones ayant un trouble mental diagnostiqué, 72,7 % ont déclaré participer à des cérémonies. La fréquentation des pensionnats (RC : 7,82) et l’expérience de la discrimination (RC: 2,69) étaient associées au fait d’avoir un trouble mental.

**Conclusion:**

OHC Toronto cherchait à combler les lacunes dans les données d’évaluation de la santé des populations autochtones en milieu urbain. Malgré leurs traumatismes présents et historiques, ces populations ont conservé leurs pratiques culturelles et un fort sentiment d’identité. Les démarches pour appuyer le bien-être des personnes autochtones doivent tenir compte des racines des traumatismes, du racisme, ainsi que des connaissances et des forces existantes des communautés autochtones.

## Introduction

In Canada, mental health and psychiatric disorders significantly affect individuals on personal and social levels. One third (33.1%) of Canadians reported symptoms of a mental health or substance use disorder in their lifetime (Statistics Canada, 2013), with most commonly reported diagnoses including anxiety disorder, major depression, bipolar disorder, and post-traumatic stress disorder (PTSD) (Statistics Canada, 2012). Mental health and psychiatric disorders often co-exist with other mental health issues (Public Health Agency of Canada, 2015), such as suicide ideation and attempt. In Canada, 11.8% reported suicidal ideation and 3.1% reported suicide attempts (Public Health Agency of Canada, 2019).

Rates of diagnosed mental health disorders are higher among Indigenous^1^ peoples than among non-Indigenous Canadians. In the 2012 Aboriginal Peoples Survey (APS), a national census-based survey of Indigenous people living in private dwellings off-reserve, 58.4% reported “excellent or very good” perceived mental health compared to 72% of the general population (Statistics Canada, 2018). Census data linked with the Canadian Institute for Health Information’s Discharge Abstract Database 2006/2007–2008/2009 demonstrated greater mental health hospitalizations per 100,000 (Ontario under-reported) for off-reserve First Nations and Métis (ratio 2.7, 2.1) than for non-Indigenous residents (Carriere et al., 2016). Suicide rates among First Nations (on- and off-reserve) and Métis (Ontario and provinces west) were triple and double non-Indigenous Canadians respectively in the 2011 Canadian Census Health and Environment Cohort (Kumar & Tjepkema, 2019; regional variability). Among APS adults, lifetime prevalence of suicidal ideation and attempts was 19.4% and 2.2%, respectively (Hajizadeh et al., 2019; significant variability among Status, Non-Status, Métis, and Inuit). The Canadian Survey on Disability (2011 National Household Survey sampling frame) and Participation and Activity Limitation Survey (2006 Census sampling frame) indicated 12.0% of Indigenous people off-reserve reported mental health and addiction disabilities compared to 4.6% of Ontarians (Ontario Human Rights Commission, 2015).

Available evidence must be interpreted with caution given varying use of mental health indicators, ethnic identifiers, geographical/political jurisdictions, and the biased, incomplete sampling frame of Indigenous surveys that draw on Census-based sampling frames. Furthermore, the Canadian Census does not include individuals living in institutions (e.g. prisons or hospitals), some First Nations on-reserve communities, collective dwellings (e.g. shelters, rooming houses, and group homes), or without a fixed address, and has a low response rate among urban Indigenous populations (Hajizadeh et al., 2019; Rotondi et al., 2017). Previous Our Health Counts projects have demonstrated that urban Indigenous people are incompletely enumerated in the Canadian Census (Rotondi et al., 2017), which is extremely problematic given that the majority of Indigenous peoples live in cities (Statistics Canada, 2017).

Poor self-rated mental health, suicidal ideation, and attempts are associated with income, housing, food and employment insecurity, and social isolation (Rotenberg, 2016; Hajizadeh et al., 2019). Conversely social inclusion, freedom from discrimination and violence (Canadian Mental Health Association, 2008), and participation in cultural continuity are important aspects of mental wellness (Chandler, 2000; Chandler & Lalonde, 2008). Cultural continuity is defined as an individual’s social and cultural connectedness to self and community as Indigenous peoples (Reading & Wein, 2009) and is considered an intermediate determinant of Indigenous peoples’ health (Reading, 2015; Reading & Wein, 2009). Cultural continuity activities include speaking Indigenous languages, and accessing traditional Indigenous medicines, foods, and ceremonies. Research has demonstrated that engagement in cultural continuity activities is a protective factor against mental health disorders (Chandler, 2000; Chandler & Lalonde, 2008), and having a sense of cultural and social connectedness (strong extended family ties, having someone to turn to for support) is more likely to have better overall health outcomes (Reading & Halseth, 2013; Rotenberg, 2016). Further research has concluded that participating in different cultural continuity activities may correlate with adaptive coping skills (Auger, 2016), and potentially moderate suicide and re-experiencing of trauma symptoms (Toombs et al., 2013).

The Truth and Reconciliation Commission of Canada (2015) described colonial policies resulting in forced relocation and loss of culture, language, and traditional social structures. Over 150,000 children attended residential schools often without parental consent, were forbidden to practice their language or culture, and experienced abuse; there are currently approximately 80,000 survivors (Truth and Reconciliation Commission of Canada, 2015). Research has shown that familial residential school attendance was associated with perceived mental health, suicidal ideation, and attempts (Hackett et al., 2016; Hajizadeh et al., 2019).

The goal of Our Health Counts (OHC) was to partner with local Indigenous communities to collect health assessment information on Indigenous peoples living in urban and related areas in Ontario, addressing the current deficiency in health information for First Nations, Inuit, and Métis living in urban and related homelands of Canada. Detailed project design can be found in other articles of this *Canadian Journal of Public Health* special issue and in previous publications (Rotondi et al., 2017). Results presented here are specific to Indigenous adult participants of the Our Health Counts Toronto study. Key objectives of this paper are to (1) understand the demographics and characteristics of Indigenous adults with a diagnosed psychological and/or mental health disorder and (2) understand potential associations of psychological/mental health diagnoses with experiences of colonization and cultural continuity among Indigenous adults in Toronto.

## Methods

### Indigenous governance and ethics

Community-specific Indigenous health research guidelines and principles informed by OCAP® (FNIGC, 2014) guided this study through the development of an Indigenous Reference Group committee and establishment of data sharing and research agreements with community partners, Seventh Generation Midwives Toronto. The Reference Group was comprised of over 25 Indigenous and allied health and social service organizations and was directly involved in all stages of project conception and implementation including specific consultations for survey tool development. Ethics approval was granted by St. Michael’s Hospital Research Ethics Board.

### Recruitment

Respondent-driven sampling (RDS) was employed to sample the Indigenous population in Toronto, Ontario. RDS is used to sample hard-to-reach populations without a sampling frame and is a modified form of snowball sampling (Heckathorn, 1997; Ramirez-Valles et al., 2005). Researchers identify *seeds* to initiate recruitment using participant’s personal networks to continue recruitment through a dual-incentive process (Heckathorn, 1997). RDS follows a Markov chain process (Ramirez-Valles et al., 2005), and continues until the final sample is independent of seeds (i.e. equilibrium is met) (Heckathorn, 1997; Ramirez-Valles et al., 2005). Indigenous people are community-oriented and relational (Castellano, 2015; Walker & Behn-Smith, 2015); therefore, using a sampling methodology that relies on social networks is useful to sample Indigenous people living in urban settings, as shown by other OHC studies in Ontario (Smylie et al., 2011).

The community partners and research team identified a heterogenous sample of 20 Indigenous adults to participate as seeds and initiate the recruitment process. Seeds were selected based on diverse characteristics and social factors, such as Indigenous identity, age, sex, occupation, marital status, parental status, and education levels in order to get a heterogenous sample. Inclusion criteria required participants to (1) self-identify as First Nation, Inuit, and/or Métis or have custody of a First Nation, Inuit, and/or Métis child 14 years of age or younger; (2) be 15 years of age or older or 14 years of age or older with a child; and (3) live, work, or access health, social, or education services in Toronto, Ontario. Data collection took place from April 2015 to March 2016 across three sites in Toronto, including Queen West Centre Toronto Community Health Centre, Seven Generations Midwives Toronto, and the Native Canadian Centre of Toronto. Home visits were also available to facilitate access to the project. A total of 915 Indigenous adults participated in the OHC Toronto project. Each participant completed a 60- to 90-min health assessment survey with a community interview using a computer-assisted web interview technique. The OHC Toronto questionnaire covered key topics including demographics, social determinants of health, physical health, access to health services, housing, and mental health.

## Measures

### Mental health diagnoses and self-rated mental health

Participants were asked “have you ever been told by a health care worker that you have a psychological and/or mental health disorder?” to assess mental health diagnoses in the population. Self-rated mental health was used to measure participant’s perceived mental health. Similar to the APS (Statistics Canada, 2018), participants were asked “in general, would you say your mental health is...” with the following options “excellent”, “very good”, “good”, “fair”, and “poor”. This variable was collapsed to include three categories: excellent/very good, good, and fair/poor.

### The Kessler Psychological Distress Scale (K-10)

The Kessler Psychological Distress Scale (K-10) was included as a mental health measure in the OHC Toronto questionnaire. K-10 has been used in previous OHC studies (Firestone et al., 2015), as well as the Aboriginal Peoples Survey (APS) (Statistics Canada, 2018) and Regional Health Survey (RHS) (First Nations Information Governance Committee, 2008). K-10 is a validated 10-item scale used to measure psychological distress and depression and anxiety symptoms in populations (Andrews & Slade, 2001). Responses to the K-10 range from “none of the time” to “all of the time” on a 5-item Likert scale, where values between 1 and 5 are assigned to responses, respectively. The sum of responses can range between 10 and 50; a score of 10 indicates no distress and a score of 50 indicates severe distress (Andrews & Slade, 2001) and scores are often categorized into the following four categories of distress: low (10–15), moderate (16–21), high (22–29), and very high (30–50).

### The Primary Care PTSD Screen (PC-PTSD)

The Primary Care PTSD Screen (PC-PTSD) was used to measure PTSD symptoms in Indigenous adults in Toronto. The PC-PTSD screening questions were included in previous OHC studies (Firestone et al., 2015). The PC-PTSD is a 4-item scale used to diagnose PTSD through screening for symptoms of PTSD, such as re-experiencing traumatic events, avoiding situations, being on guard and easily startled, and/or feeling numb/detached from others and surroundings. Participants who respond “yes” to a minimum of 3 of the 4 PTSD indicators are considered to have a positive PTSD diagnosis (Prins et al., 2003).

### Cultural continuity and social connectedness measures

Inspired by Chandler and Lalonde’s research exploring the association between cultural continuity and youth suicide in First Nations communities in British Columbia (Chandler & Lalonde, 1998; Chandler & Lalonde, 2008), four measures from the OHC Toronto questionnaire were used to understand the impact of participation in cultural continuity activities on mental health diagnosis. Participants were asked the following three binary questions “do you participate in traditional Indigenous ceremony”, “do you speak an Indigenous language”, and “do you use traditional Indigenous medicines or practices to maintain your health and wellbeing”, with responses being “yes” or “no”. To assess traditional food consumption, participants were asked “in the past 12 months, how often have you eaten traditionally hunted/gathered/grown or country foods” with possible responses including “often”, “a few times”, or “not at all”. Social connectedness was measured using the question “about how many close friends and close relatives do you have, that is people you feel at ease with and can talk to about what is on your mind” with possible responses ranging from “0” to “more than 10”. Both of these categorical responses were collapsed to have a binary classification with “yes” being more than 10 and “no” being 0 to 10.

### Measures of family disruption linked to colonial policies

Modified from the personal and familial residential school attendance measures on the Regional Health Survey (First Nations Information Governance Committee, 2008), OHC participants were asked “were you ever a student at a federal residential school, or federal industrial school” to assess whether participants attended residential schools. To determine whether a participant had family members attend residential schools, participants were asked “were any of the following members of your family ever a student at a federal residential school or a federal industrial school”. Participants had the opportunity to select from a list of family members such as great-grandparents, grandparents, parents, siblings, spouse, and cousins. Responses were collapsed to have a binary classification. To determine whether participants were affected by the Sixties Scoop, participants were asked “were you or other members of your family adopted between 1951 to 1970, during the Sixties Scoop”. Both questions had “yes” or “no” response options.

### Measures of racism

Experiences of anti-Indigenous discrimination were measured using the question “have you ever been treated unfairly because you are Aboriginal”.

### Statistical analysis

RDS-II weights were applied to calculate population-based estimates with 95% confidence intervals using the RDS Package in R Software (R Core Team, 2013) and odds ratios with 95% confidence intervals using SAS 9.4 Software (SAS Institute Inc., 2013). Odds ratios were calculated to measure the association between mental health diagnosis and experiences of colonization. RDS-II weights were calculated using the inverse of participant’s network sizes and are applied to analyses to account for the unequal probability of recruitment due to varying network sizes among participants (Salganik & Heckathorn, 2004).

## Results

### Mental health

Among the 915 Indigenous adults in Toronto, 45.0% (95% CI: 39.4, 50.6) reported having a psychological/mental health disorder as diagnosed by a health care provider. Among those with a diagnosis, the majority reported having an anxiety (53.3%, 95% CI: 44.4, 62.2) and/or major depression (50.8%, 95% CI: 41.8, 59.7) diagnosis.

### Characteristics of Indigenous adults with a diagnosed psychological or mental health disorder

Among Indigenous adults with a diagnosed psychological or mental health disorder, 55.5% identified as female (95% CI: 46.6, 64.5) and 43.6% as male (95% CI: 34.7, 52.6), and were between the ages of 26 and 54 (75.7%, 95% CI: 68.2, 83.3) and were single (65.5%, 95% CI: 57.2, 73.8). The majority were unemployed (70.5%, 95% CI: 61.9, 79.0) and living below the low-income cut-off (87.3%, 95% CI: 81.2, 93.3). Most reported having educational attainment of high school or less (49.8%, 95% CI: 40.9, 58.8). The majority reported having stable housing (52.7%, 95% CI: 44, 61.4) (Table 1).

**Table 1:** Demographics and characteristics of OHC Toronto adults in Toronto, Ontario with and without a diagnosed psychological or mental health disorder

Characteristics	Indigenous adults in Toronto (*n*=915)		Indigenous adults with diagnosed psychological or mental health disorder (*n*=375)		Indigenous adults with no diagnosed psychological or mental health disorder (*n*=535)	
	*n*	RDS-II % (95% confidence interval)	*n*	RDS-II % (95% confidence interval)	*n*	RDS-II % (95% confidence interval)
Indigenous identity						
First Nations	804	85.5 (81.5, 89.5)	317	86.5 (80.9, 92.1)	483	84.9 (79.3, 90.5)
Métis	89	13.4 (9.6, 17.3)	49	12.0 (6.7, 17.2)	39	14.4 (8.8, 20.0)
Inuit	11	0.4 (0.0, 0.9)	-	0.4 (0.0, 0.7)	-	0.5 (0.0, 1.3)
Multiple Indigenous identities	8	0.5 (0.0, 1.3)	-	0.9 (0.0, 2.9)	-	0.1 (0.0, 0.2)
Other	-	0.2 (0.0, 0.5)	-	0.5 (0.0, 1.3)	-	0.0 (0.0, 0.)
Age						
Under 25 years	126	23.3 (18.6, 28.0)	28	18.6 (12.0, 25.1)	87	27.3 (20.9, 33.8)
26 to 54 years	603	63.0 (57.4, 68.5)	296	75.7 (68.2, 83.3)	314	52.2 (44.8, 59.6)
55+ years	186	13.8 (9.5, 18.1)	51	5.7 (1.0, 10.4)	134	20.4 (14.1, 26.8)
Gender						
Male	421	49.7 (44.0, 55.3)	150	43.6 (34.7, 52.6)	266	54.2 (46.8, 61.5)
Female	477	48.9 (43.1, 54.5)	218	55.5 (46.6, 64.5)	259	43.9 (36.5, 51.2)
Other	-	0.4 (0.0, 1.5)	-	0.5 (0.0, 1.3)	-	0.4 (0.0, 2.1)
Trans	12	1.0 (0.3, 1.7)	-	0.4 (0.0, 1.4)	-	1.4 (0.6, 2.4)
Income (low-income cut-off)						
Above	188	13.1 (9.3, 16.9)	67	12.8 (6.7, 18.8)	121	13.5 (8.5, 18.4)
Below	713	86.9 (83.1, 90.7)	305	87.3 (81.2, 93.3)	406	86.5 (81.6, 91.5)
Employment status						
Employed	273	18.2 (13.6, 22.8)	92	15.1 (7.9, 22.3)	181	21.0 (14.9, 27.0)
Unemployed	473	63.0 (57.4, 68.6)	232	70.5 (61.9, 79.0)	236	56.4 (48.9, 63.8)
Not in labour force	135	18.8 (14.3, 23.3)	39	14.4 (8.2, 20.7)	96	22.7 (16.5, 28.9)
Education level						
Some high school or less	390	49.4 (43.7, 55.1)	151	49.8 (40.9, 58.8)	236	49.0 (41.5, 56.5)
Completed high school	171	17.9 (13.2, 22.7)	72	12.3 (6.2, 18.4)	98	22.3 (15.5, 29.1)
Some or completed college	226	24.8 (20.1, 29.5)	103	30.1 (22.0, 38.2)	122	20.7 (15.1, 26.3)
Some or completed university	126	7.8 (4.8, 10.8)	49	7.8 (2.3, 13.3)	77	8.0 (4.6, 11.4)
Housing						
Stable housing	664	64.9 (59.8, 70.0)	257	52.7 (44.0, 61.4)	406	75.1 (68.9, 81.2)
Precarious housing	70	7.8 (4.9, 10.7)	31	5.8 (1.5, 10.0)	39	9.2 (5.3, 13.0)
Homeless	154	27.3 (22.7, 31.9)	76	41.6 (33.3, 49.9)	76	15.8 (10.6, 20.9)
Marital status						
Single	565	64.4 (59.0, 69.8)	230	65.5 (57.2, 73.8)	330	63.2 (56.0, 70.4)
Girlfriend/boyfriend	137	14.8 (11.0, 18.5)	60	15.5 (10.1, 21.0)	77	14.3 (9.2, 19.4)
Common law/cohabitating	124	13.1 (8.9, 17.4)	51	11.1 (4.5, 17.7)	73	14.9 (9.3, 20.5)
Married and cohabitating	50	4.1 (2.2, 6.0)	14	3.1 (9.7, 5.5)	36	5.0 (2.3, 7.7)
Separated	34	3.6 (1.6, 5.5)	18	4.8 (1.9, 7.8)	16	2.6 (0.0, 5.2)

Most Indigenous adults with a diagnosed psychological or mental health disorder rated their mental health as good (48.5%, 95% CI: 39.6, 57.3) or fair/poor (39.1%, 95% CI: 30.5, 47.6) and had very high (36.3%, 95% CI: 27.4, 45.2) or high (29.4%, 95% CI: 21.4, 37.5) K-10 scores. Approximately half had three or more PTSD symptoms (49.8%, 95% CI: 40.9, 58.7). There were extremely high rates of suicide ideation (77.8%, 95% CI: 69.8, 85.7), as well as high rates of self-harm and suicide attempt (Table 2).

**Table 2:** Characteristics of OHC Toronto adults in Toronto, Ontario with and without a diagnosed psychological or mental health disorder

Characteristics	Indigenous adults in Toronto (*n*=915)		Indigenous adults with diagnosed psychological or mental health disorder (*n*=375)		Indigenous adults with no diagnosed psychological or mental health disorder (*n*=535)	
	*n*	RDS-II % (95% confidence interval)	*n*	RDS-II % (95% confidence interval)	*n*	RDS-II % (95% confidence interval)
Mental wellness rating						
Excellent/very good	300	31.5 (26.1, 37.0)	60	12.5 (5.9, 19.0)	239	47.1 (39.7, 54.5)
Good	347	40.0 (34.5, 45.5)	146	48.5 (39.6, 57.3)	201	33.0 (26.1, 40.0)
Fair/poor	261	28.5 (23.5, 33.5)	167	39.1 (30.5, 47.6)	94	19.9 (14.0, 25.7)
Kessler						
Low	308	27.4 (22.0, 32.8)	63	13.1 (6.6, 19.6)	245	37.7 (30.4, 45.1)
Moderate	229	22.4 (17.7, 27.0)	87	21.2 (13.9, 28.5)	142	23.2 (17.2, 29.2)
High	210	30.8 (25.6, 35.9)	111	29.4 (21.4, 37.5)	99	31.7 (25.0, 38.5)
Very high	145	19.5 (14.8, 24.1)	106	36.3 (27.4, 45.2)	38	7.3 (3.1, 11.6)
PTSD						
Nightmares	328	37.1 (31.6, 42.7)	186	54.2 (45.2, 63.1)	142	23.6 (17.0, 30.3)
Avoided trauma	397	46.0 (40.3, 51.7)	22	61.0 (52.6, 69.3)	172	33.7 (26.7, 40.7)
Startled by trauma	361	40.5 (34.9, 46.1)	211	58.3 (49.7, 66.9)	149	25.9 (19.4, 32.4)
Detached/numb from others or surroundings	333	40.6 (35.0, 46.1)	202	59.1 (50.5, 67.7)	131	25.4 (19.1, 31.7)
3 or more	295	31.0 (25.7, 36.3)	188	49.8 (40.9, 58.7)	107	16.0 (10.3, 21.7)
Suicide						
Family/friend suicide	506	58.0 (52.3, 63.7)	235	68.2 (59.4, 76.9)	270	49.6 (42.1, 57.0)
Self-harm	345	46.6 (41.0, 52.2)	209	64.8 (56.1, 73.6)	135	30.1 (23.8, 37.5)
Suicide ideation	454	54.3 (48.6, 60.1)	266	77.8 (69.8, 85.7)	186	33.9 (27.1, 40.7)
Suicide attempt	279	37.3 (32.2, 42.3)	209	55.5 (46.5, 64.4)	97	21.3 (15.4, 27.1)

### Cultural continuity and social connectedness

There were high rates of participation in cultural continuity activities such as speaking Indigenous languages, using traditional Indigenous medicines, eating traditional Indigenous foods, and participating in ceremony among Indigenous adults living in Toronto. Of Indigenous adults with a diagnosed psychological or mental health disorder, 46.8% reported speaking one or more Indigenous languages (95% CI: 38, 55.5), 51.9% used traditional Indigenous medicines (95% CI: 42.8, 60.9), 72.7% reported participating in ceremony (95% CI: 64, 81.4), and 52.6% ate traditional Indigenous foods (95% CI: 43.5, 61.6). The majority reported having a sense of social connectedness (i.e. a close friend or relative they could talk to about what is on their mind) (Table 3).

**Table 3:** Cultural continuity activities and social connectedness among OHC Toronto adults with and without a diagnosed psychological or mental health disorder

Characteristics	Indigenous adults in Toronto (*n*=915)		Indigenous adults with diagnosed psychological or mental health disorder (*n*=375)		Indigenous adults with no diagnosed psychological or mental health disorder (*n*=535)	
	*n*	RDS-II % (95% confidence interval)	*n*	RDS-II % (95% confidence interval)	*n*	RDS-II % (95% confidence interval)
Speak Indigenous language(s)						
Yes	438	41.2 (35.7, 46.7)	173	46.8 (38.0, 55.5)	263	36.6 (29.5, 43.6)
No	477	58.8 (53.3, 64.3)	202	53.3 (44.5, 62.0)	272	63.4 (56.4, 70.5)
Participate in ceremony						
Yes	693	65.0 (59.3, 70.7)	294	72.7 (64.0, 81.4)	397	58.6 (51.2, 66.1)
No	219	35.0 (29.3, 40.7)	81	27.3 (18.6, 36.0)	138	41.4 (33.9, 48.8)
Use of traditional Indigenous medicines						
Yes	565	49.6 (43.9, 55.2)	253	51.9 (42.8, 60.9)	312	47.9 (40.5, 55.2)
No	346	50.4 (44.8, 56.1)	122	48.1 (39.1, 57.2)	222	52.1 (44.8, 59.5)
Eat traditional Indigenous foods						
Yes	537	50.5 (44.8, 56.2)	216	52.6 (43.5, 61.6)	320	48.8 (41.3, 56.2)
No	374	49.5 (43.8, 55.2)	157	47.4 (38.4, 56.5)	214	51.2 (43.8, 58.7)
Social connectedness						
Yes	822	91.1 (87.4, 94.7)	332	93.4 (88.4, 98.5)	487	89.0 (84.0, 94.1)
No	91	8.9 (5.3, 12.6)	43	6.6 (1.2, 11.6)	48	11.0 (5.8, 16.0)

### Experiences of colonization and racism

Controlling for age and gender, attending residential schools was associated with having a psychological or mental health disorder diagnosis (OR: 3.3, 95% CI: 1.0, 10.6) and experiencing discrimination was associated with having a psychological or mental health disorder diagnosis (OR: 2.8, 95% CI: 1.5, 5.3) (Table 4).

**Table 4:** RDS-II adjusted odds ratios relating experiences of colonization to diagnosed psychological or mental health disorder among OHC Toronto adults

Characteristics	Indigenous adults in Toronto (*n*=915)		Indigenous adults with diagnosed psychological or mental health disorder (*n*=375)		Indigenous adults with no diagnosed psychological or mental health disorder (*n*=535)		RDS-II odds ratio (95% confidence interval)
	*n*	RDS-II % (95% confidence interval)	*n*	RDS-II % (95% confidence interval)	*n*	RDS-II % (95% confidence interval)	
Residential school attendance							
Yes	76	10.4 (7.3, 13.5)	33	14.5 (9.0, 19.9)	42	6.6 (3.1, 10.1)	3.3 (1.0, 10.6)*
No	830	89.6 (86.5, 92.7)	340	85.5 (80.1, 91.0)	488	93.4 (89.9, 96.9)	
Intergenerational (family) residential school attendance							
Yes	596	58.5 (52.9, 64.1)	246	64.6 (55.8, 73.4)	348	53.7 (46.4, 61.0)	1.63 (0.9, 3.1)
No	319	41.5 (35.9, 47.1)	129	35.4 (26.7, 44.2)	187	46.3 (39.0, 53.6)	
Sixties Scoop							
Yes	270	24.6 (19.5, 29.8)	127	31.3 (22.3, 40.4)	143	19.5 (13.5, 25.5)	1.9 (1.0, 3.8)
No	573	75.4 (70.2, 80.6)	211	68.7 (59.7, 77.7)	359	80.6 (74.6, 86.6)	
Experience of discrimination							
Yes	570	53.8 (48.0, 59.5)	268	67.2 (58.3, 76.1)	301	43.2 (35.9, 50.6)	2.8 (1.5, 5.3)*
No	335	46.2 (40.5, 52.0)	103	32.8 (24.0, 41.7)	231	56.8 (49.4, 64.1)	

*Refers to a statistically significant association

All odds ratio estimates were RDS-II adjusted and controlled for age and gender

## Discussion

The goal of this research was to work in partnership with Indigenous communities to address the current deficiency in Canadian urban Indigenous health data by generating a representative sample of Indigenous people living in an urban setting. Through the successful implementation of RDS, population estimates and 95% confidence intervals were produced for mental health, cultural continuity, and experiences of colonization indicators. Adjusted odds ratios relating experiences of colonization to diagnosed psychological or mental health disorder were also generated. Our findings confirm existing community concerns, highlighting the elevated levels of emotional suffering experienced by this population as well as community strengths and connectedness as measured by participation in cultural continuity activities such as speaking Indigenous languages, using traditional Indigenous medicines, eating traditional Indigenous foods, and participating in ceremony and having a sense of social connectedness.

It is important to recognize that in addition to high rates of diagnosed mental health disorders, many Indigenous adults in Toronto also reported good levels of self-rated health; felt in balance of the physical, mental, emotional, and spiritual aspects of self; and exhibited a strong sense of cultural identity through high participation in cultural practices. Cultural continuity and social connectedness have been explored as protective factors against substance use, suicide ideation and suicide attempts, depressive symptoms, and chronic PTSD in Indigenous settings (Chandler & Lalonde, 1998; Chandler & Lalonde, 2008). Our findings resonate with emerging research that culture and culture-based coping mechanisms are a key modality of maintaining wellness for Indigenous peoples (Gone, 2013; Gray & Cote, 2019; Rowan et al., 2014). Furthermore, the Our Health Counts Toronto data reinforce growing evidence for mental health and substance use treatment models that involve integrated, holistic community, and culture-based approaches (Rowan et al., 2014).

In the context of ongoing colonial policies, persistent impacts of historic trauma, and socio-economic marginalization, Indigenous people suffer from elevated burdens of mental health disorders. Almost all of the Indigenous adults in Toronto who were diagnosed with a mental health or psychological disorder were living below the low-income cut-off. Also, Indigenous adults were significantly more likely to have a mental health diagnosis if they had attended residential school or had been treated unfairly because of their Indigenous identity. These findings demonstrate the intersectionality between experiences of trauma, family disruption, and discrimination, shedding light on the contextual and systemic factors that contribute to emotional suffering, anxiety, and depression. While Indigenous-led approaches such as Indigenous midwifery and doula care (Churchill, 2015), Indigenous peer support initiatives (MacDonald, 2019), Indigenous child protection (Institute for Urban Indigenous Health, 2014) and Indigenous Narrative Exposure Therapy (Smylie et al., 2020) exist in Canada and abroad, significant challenges in access to culturally secure, trauma-informed health services persist.

### Limitations

RDS methodologies are still evolving and there are still statistical limitations with RDS data analysis. Due to the social network nature of RDS, non-random sampling bias is created because individuals with larger social networks are more likely to be recruited to participate in an RDS study. RDS-II weights were employed to adjust for this bias. While there may have been some limitations with self-reporting and response rates, particularly around experiences and behaviours that are stigmatizing or too painful to recall, all interviews were conducted by members of the community in a safe and neutral place and interviewers provided continued assurance of confidentiality. Although minimal in the Our Health Counts study, low response rates are not accounted for in RDS methodologies.

Rates of mental health diagnoses may be underestimated due to undiagnosed mental health conditions and barriers to accessing health care providers who can make diagnoses. In addition, the impacts of colonization are likely underestimated due to the long-standing nature of colonial policies against Indigenous peoples in Canada. There is no way of measuring the impact of colonization in its entirety with the limited information that was provided in the OHC questionnaire.

## Conclusion

Findings from Our Health Counts Toronto support existing national recommendations (Truth and Reconciliation Commission of Canada, 2015) that municipal, provincial, and federal government ensure the provision of adequate funding to urban Indigenous child, youth, and adult mental health strategies and programs. The governance, design, implementation, and translation of Indigenous health services and programs must be led by Indigenous people and informed by health information that accurately reflects Indigenous knowledge and experiences. Through OHC, we have identified the gaps and inequities in mental health outcomes as well as directions for closing these gaps by addressing physical, emotional, spiritual, and mental harms experienced by Indigenous people in this country.

## Contributions to knowledge

What does this study add to existing knowledge?

- This study has generated population estimates for mental health, cultural continuity, and experiences of colonization and racism among the Indigenous adults in Toronto.
- Culture and culture-based coping mechanisms can be protective factors amid high burdens of mental health disorders and historic and ongoing trauma.

What are the key implications for public health interventions, practice, or policy?

- Findings reinforce TRC recommendations including municipal, provincial, and federal funding for urban Indigenous mental health strategies and programs.
- Indigenous health services and programs must be governed and delivered by Indigenous people and informed by accurate health information.

## Data Availability

N/A

## Footnotes

1 “Indigenous” is a collective term including the original peoples of Canada and their descendants (“Aboriginal” is often used). The 1982 *Constitution Act* includes Indian (referred to as “First Nations”), Métis, and Inuit (Statistics Canada, 2017). For purposes of the Our Health Counts project, individuals were able to self-identify as First Nations, Inuit, or Métis regardless of whether they are registered under the Indian Act, registered with a provincial Métis organization, or a beneficiary of an Inuit land claim.

## Code availability

N/A
